# Distinctive prokaryotic microbiomes in sympatric plant roots from a Yucatan cenote

**DOI:** 10.1186/s13104-021-05746-x

**Published:** 2021-09-07

**Authors:** Alejandra Escobar-Zepeda, Patricia Rosas-Escobar, Laura Marquez Valdelamar, Patricia de la Torre, Laila P. Partida-Martinez, Ruben Remegaldo, Alejandro Sanchez-Flores, Fredd Vergara

**Affiliations:** 1 Instituto de Biotecnologia, Unidad Universitaria de Secuenciacion Masiva Y Bioinformatica, Universidad Nacional Autonoma de Mexico, Cuernavaca, Mexico; 2 LaNaBio, Instituto de Biologia, UNAM, Mexico City, Mexico; 3 Instituto de Investigaciones Biomedicas, UNAM, Mexico City, Mexico; 4 Departamento de Ingenieria Genetica, Centro de Investigacion Y de Estudios Avanzados, Irapuato, Mexico; 5 Macroecology and Society, German Centre for Integrative Biodiversity Research Halle-Jena-Leipzig, Leipzig, Germany; 6 Molecular Interaction Ecology/EcoMetEoR, German Centre for Integrative Biodiversity Research Halle-Jena-Leipzig, Leipzig, Germany; 7 Institute of Biodiversity, Friedrich Schiller University Jena, Jena, Germany

**Keywords:** 16S *r*RNA, Cenote, *Ficus obtusifolia*, *Gliricidia sepium*, Taxonomic profiling, Rhizosphere, *Trichilia hirta*

## Abstract

**Objective:**

Cenotes are flooded caves in Mexico’s Yucatan peninsula. Many cenotes are interconnected in an underground network of pools and streams forming a vast belowground aquifer across most of the peninsula. Many plants in the peninsula grow roots that reach the cenotes water and live submerged in conditions similar to hydroponics. Our objective was to study the microbial community associated with these submerged roots of the Sac Actun cenote. We accomplished this objective by profiling the root prokaryotic community using 16S *r*RNA gene amplification and sequencing.

**Results:**

We identified plant species by DNA barcoding the total genomic DNA of each root. We found a distinctive composition of the root and water bacterial and archaeal communities. Prokaryotic diversity was higher in all plant roots than in the surrounding freshwater, suggesting that plants in the cenotes may attract and select microorganisms from soil and freshwater, and may also harbor vertically transmitted lineages. The reported data are of interest for studies targeting biodiversity in general and root-microbial ecological interactions specifically.

**Supplementary Information:**

The online version contains supplementary material available at 10.1186/s13104-021-05746-x.

## Introduction

**Fig. 1:**
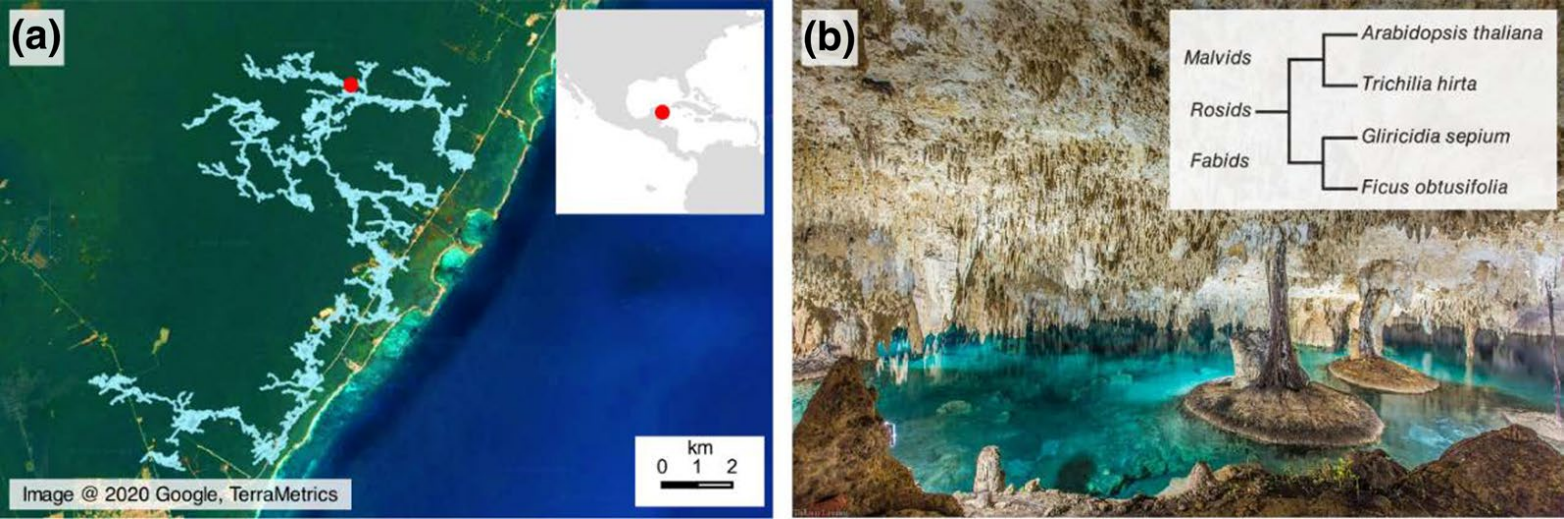
Sampling site in the Yucatan peninsula, Mexico. (**a**) The location of the Sac Actun cenote is highlighted in red and the currently surveyed extension of the local underground cave system is shown in blue [15]. The high-resolution basemap was obtained through Google earth and the continental delineation originates from the Database of Global Administrative Areas v3.6 (GADM). (**b**) Photograph of the sampling site (https://search.creativecommons.org/photos/fd1d5a63-ae74-457a-944b-a4c3521c232f). The insert shows a cladogram with the phylogenetic relationship of the plant species investigated in reference to *A*. *thaliana*. We used DNA barcoding for plant species identification

The Yucatan peninsula in Southeastern Mexico presents a characteristic landscape devoid of high mountains and aboveground rivers. The peninsula is a partially emergent carbonate platform where Mesozoic- and Cenozoic-era limestone, dolomite, and anhydrite overlie deeply buried Paleozoic-era crystalline and sedimentary rocks [1]. The main inland ecosystems are evergreen and deciduous tropical forests [2] and the mean precipitation in the peninsula is approx. 1200 mm per annum [3] (Fig. 1). Rainwater rapidly percolates and dissolves the porous, karstic bedrock forming large cavities [4]. In some cases, the walls of these caves collapse leaving behind sinkholes locally known with the Mayan name of cenotes. Rainwater accumulates in the cenotes, many of which are interconnected in an underground network of pools and streams that form a vast belowground aquifer across most of the peninsula [5]. The water contained in the cenotes is the only year-round reliable source of fresh water in this region and thus, the cenotes have influenced the development of human civilization, but also other species including plant communities [6]. In the case of plants, roots penetrate the cenotes’ ceiling rocks, which sometimes can be several meters wide, to reach freshwater reservoirs inside the cenotes. These roots remain immersed in the freshwater, resembling a hydroponic system.

Bacterial microbiomes and their role in plant roots have been extensively studied in terrestrial ecosystems. For example, in terrestrial-only ecosystems, plant roots interact with a multitude of microorganisms in the soil, which provide nutrients and are involved in nitrogen fixation. Also, root associated soil microbial communities participate in plant nutrition, development and immunity, as well as in tolerance to several types of biotic and abiotic stresses. Correspondingly, the plant innate immune system must simultaneously tolerate beneficial microbes to survive while limiting the growth of potential pathogens [7]. However, for plants with steady water-immersed roots, the microbial-root interaction remains understudied. The data we provide in this report contributes to expand our knowledge of root microbe interactions under in a freshwater environment.

## Main text

### Materials and methods

#### Sample collection and pre-processing

Roots and fresh water from three plants were collected in the Sac Actun cenote (20°19′49″ N 87°24′13″ W) in Yucatan, Mexico in April, 2013. Roots were not in physical contact with each other and separated 5 to 10 m. Roots were collected at a depth of 30 to 50 cm from the air/water interface. Samples were immediately placed on ice and air-transported to Universidad Nacional Autonoma de Mexico in Mexico City. Upon arrival, samples were freeze-dried and kept at − 80 °C until further processing.

#### Plant species identification by DNA barcode

Total genomic DNA was isolated from freeze-dried roots using the Qiagen Dneasy plant kit following manufacturer’s protocol. All amplifications were performed in a 2720 thermal cycler (Applied Biosystems) with 40–50 ng of DNA, 1 × PCR buffer, 2 mM MgCl_2_, 1.6 µg bovine serum albumin (BSA), 200 µM of each dNTP, 0.12 µM of each primer and 0.625 U of Taq Platinum polymerase (Invitrogen) in 25 µL total volume. Primers recommended by the Plant Working Group of the Consortium for the Barcode of Life (http://www.boldsystems.org) were used to amplify four different genome regions (Additional file 3: Table S2). Amplicons were confirmed by electrophoresis on a 1% agarose gel and further purified with sephadex columns (GE Healthcare Life Sciences) following user’s manual. Purified PCR products were bi-directionally sequenced using the original PCR primers with the BigDye v3.1 on an ABI 3500xL genetic analyzer (Applied Biosystems). Sequences were pre-processed with Sequencing Analysis Software v6.0 (Applied Biosystems) and further edited and assembled with Sequencher v5.0.1 (Gene Codes Corporation). Individual sequences were blasted against databases hosted in the National Center for Biotechnology Information (NCBI) and the Barcode of Life Data Systems (BOLD) for species identification. For plant species identification, a total of 553 bp were obtained for the *rbcL* region, 808 bp for *matK* and 402 bp for *trnH*-*psbA*. For the first sample, BLAST results obtained for the three regions showed the most similar genus was *Ficus* (Moraceae). However, it was not possible to infer the species because 99% identity resulted in more than one species, but using only the *trnH*-*psbA* marker, we obtained 100% of identity hit to *Ficus obtusifolia*. For the second sample for *rbcL* a total of 100% of similarity was found for *Trichilia* (Meliaceae) and 99% to *Dysoxylum*, *Heckeldora*, *Leplaea*, *Chisocheton*, *Turreanthus*, *Walsura*, *Vavaea* and *Guarea* genera, but these genera are not reported for Mexico. Also, for *matK* 99% of similarity was found for *Trichilia*. To achieve species level identification, a sample of two specimens (*T*. *hirta*: MEXU 1,167,324 and *T*. *havanensis*: MEXU 1,024,025) from National Herbarium of Mexico (MEXU) were used. DNA of these samples was extracted using standard method for plants [8]. *rbcL* and *matK* regions were amplified using primers in Additional file 3: Table S2 and sequenced. Comparing the sample against *T*. *hirta* and *T*. *havanensis*, 100% of identity was found against *T*. *hirta*. For the third sample, BLAST results for *rbcL* and *matK* markers reported 100% of identity to *Gliricidia sepium* (Fabaceae), and 99% of similarity for *G*. *sepium* and *G*. *maculate*, respectively. For the latter marker, both result names are synonymous, so the species was considered as *G*. *sepium*. Cladogram in Fig. 1 was generated based on the plant lineages using the ETE3 package [9]. GenBank accession numbers are shown in Additional file 3: Table S3.

#### 16S *r*RNA sequencing using Ion Torrent Technology

For DNA isolation, the roots were macerated in 50 ml of sterile distilled water. Subsequently, the supernatant was passed through a 0.45 µm filter (Merck Millipore Corporation). With this filtrate, DNA extraction was continued using the Power Water DNA isolation kit (MoBio Laboratories Inc., Carlsbad, CA, USA). DNA quality and quantity was evaluated by electrophoresis and using a Nanodrop (1000) (Thermo Scientific). 16S *r*RNA gene amplification was carried out using the Ion 16S™ Metagenomics Kit (Life Technologies) following the manufacturer's protocol using two primer pools to amplify seven hypervariable regions (V2, V3, V4, V6, V7, V8, and V9) of bacterial 16S *r*RNA. 2 µL of each sample were needed along with the Ion Xpress Barcode Adapters 1–16 Kit (Life Technologies, Carlsbad, CA, USA). For library construction the Ion Plus Fragment Library kit (Life Technologies) was used following manufacturer protocol. Quality of samples was evaluated in an Agilent Bioanalyzer 2000 with Agilent High Sensitivity DNA kit (Agilent Technologies). Library concentration was measured with a qPCR using the Ion Universal Library Quantitation Kit in a Step One Real-Time PCR System (Applied Biosystems). Samples were adjusted to 10 mM final concentration. PCR was conducted on an Ion One Touch 2 using the Ion PGMTM Template OT2 400 (Life Technologies), and Sequencing on an Ion PGM using an Ion 310 v2 chip, following manufacturer’s protocol.

#### 16S *r*RNA sequences processing and analyses

**Fig. 2:**
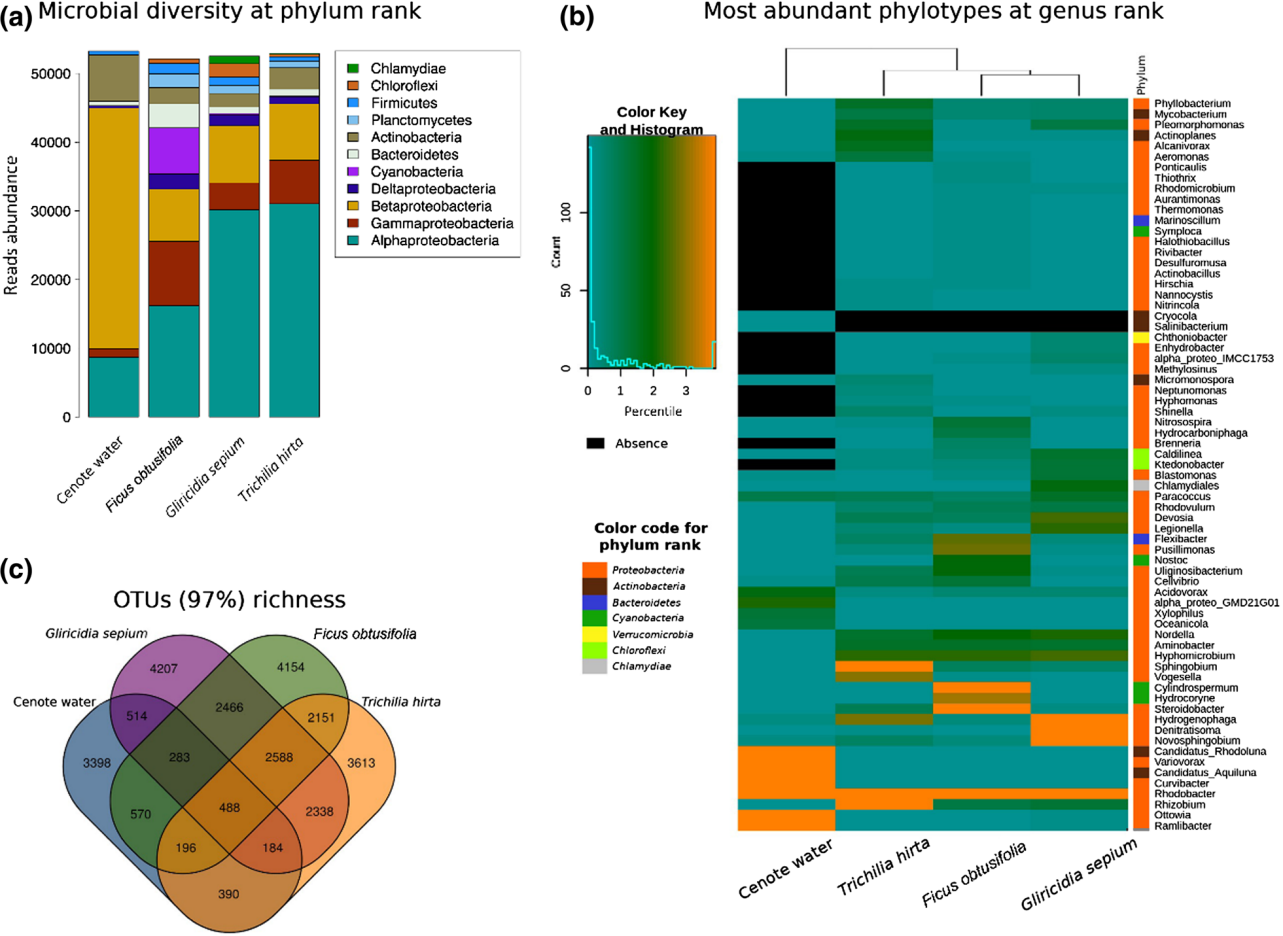
Structure of the root microbial communities from plant roots and freshwater collected in April 2013 from the Sac Actun cenote in the Yucatan peninsula, Mexico. (**A**) Reads abundance by sequence identity to prokaryotic references depicted at phylum rank. Low abundance phyla list is available in Additional file 2: Table S1. (**B**) Comparison of the most abundant genera found in cenote water and plant roots. The color scale represents the genera abundance distribution in percentiles. (**C**) Venn diagram representing unique and shared OTUs (97%) between samples of water and plant roots

For bacterial taxonomic profiling, the 16S *r*RNA amplicon reads raw reads were analyzed using Perl scripts (https://github.com/Ales-ibt/16S_processing). Sequences were filtered by length < 50 bp and identical sequences were dereplicated through the –derep_fulllength function from VSEARCH tool v2.4.3 [10]. Unique sequences were clustered at 97% of sequence identity (OTUs0.03) by the –cluster_fast function. To discard artificial diversity, we followed a filtering strategy of chimeric sequences detection using the –uchime_denovo function. Additionally, the clusters of size 1 represented in one sample only were discarded from the OTU table, as well as all those sequences which annotation did not match to Bacteria or Archaea domains. The final OTU table was rarefied to 53,659 reads using the rrarefy function from the R Vegan library v2.4–6 to calculate alpha diversity. We calculated the Chao1 richness estimator and the Shannon diversity index using the R Phyloseq library [11]. The Good’s coverage was estimated using a Perl script according to the formula: 1-(singletons/total reads). Sequences were taxonomically labeled following a sequence identity strategy by Blastn with the Megablast parameters against a database based on SILVA and curated with RDP and GG, which is available with the Metaxa2 v2.1.1 [12] software distribution. Taxonomic annotation was transferred to each of the representative sequences from rarefied OTUs (97%). The heatmap in Fig. 2B was constructed in R using the heatmap2 function from gplots v3.0.1.1 library.

### Results

The cenote’s root bacterial communities, which include in our study the root episphere and the root endosphere of each of the characterized plant species, showed similar Chao and Shannon diversity indexes which both were higher than those observed in the cenote freshwater (Table 1). Beta diversity analysis showed that the cenote water microbial community is an outgroup in relation to the cenotes roots cluster. Within the latter, the *Ficus obtusifolia* microbial community was the most dissimilar (Additional file 1: Figure S1).

**Table 1:** Analysis of bacterial and archaeal OTUs diversity in roots and in the cenote’s water

Sample	Total reads	Average read length (bp)	Prokaryotic OTU’s (97%)	Chao1	Sampling effort	Shannon index	Good’s coverage
Cenote water	104,722	209.79	6,023	8,549	70.45	5.75	0.951
*Gliricidia sepium*	136,573	184.96	12,896	19,838	65.01	8.23	0.884
*Ficus obtusifolia*	131,302	171.42	13,068	17,824	73.32	8.37	0.896
*Trichilia hirta*	112,632	178.77	11,948	17,253	69.25	7.99	0.899

A deeper analysis of shared taxa between water and plant-associated microbial communities, revealed 2588 OTUs common to all three roots that were absent in the cenote freshwater. Only 488 OTUs were also shared among the cenote water and all plant roots (Fig. 2). The OTUs present in roots represented 219 distinct genera from 107 families, sustaining the observation of greater microbial diversity. Despite these high number of shared OTUs (2588 out of 27,541 or 9.40%), the relative abundance varied in sampled plant roots (Fig. 2). These results reinforce the idea that microbiome found in plant roots immersed in aquatic ecosystems are influenced by the host.

Increasing evidence shows that root exudates recruit, nurture or repel different types of microorganisms in soil-based rhizospheres [13, 14]. However, information of aquatic root microbial communities and knowledge regarding how these are shaped in aquatic environments remain scarce. We investigated the composition and diversity of the bacterial and archaeal communities colonizing the roots of three sympatric plant species living immersed in the freshwater of the cenotes by high-throughput taxonomic profiling. This survey provides data helping botanists and microbiologist better understand root/microbiota interactions in non-classical environments.

### Conclusions

Studies of the ecological effects of root microbiomes in plants had been primarily performed in soil environments. Consequently, the cenotes offer a unique opportunity to expand our understanding of root-microbial associations beyond those interactions occurring in most terrestrial ecosystems. Our finding that each root microbiome analysed here has a distinct and highly diverse microbial community profile, that is not shared with the one from the surrounding freshwater, motivates further investigations. Future comparisons should include comparisons of root microbiomes of the same host species in purely terrestrial ecosystems, as well as in the cenotes, with the purpose of distinguishing the effects of host selection from those imposed by the habitat (soil vs. aquatic). Moreover, separation of “rhizosphere” from “root endosphere” in this ecosystem may facilitate the identification of microbial groups that clearly respond to root exudates and those that may be of vertical transmission. Finally, evaluation of the fungal communities associated with the roots could also shed light on the role of fungal symbionts, such as mycorrhizal fungi, in the adaptation of plants to this unique niche. In summary, our data strongly suggest that sympatric plants roots living immersed in the freshwater of cenotes harbor a distinct and highly diverse prokaryotic community, which seems to be influenced by the host and its hydroponic-like growing conditions.

## Limitations

One limitation of our study was the lack of biological replicates, which are necessary for quantitative estimations of host selection and microbial diversity. However, finding biological replicates in the unique niche of the cenotes is extremely difficult. Since there is no indication aboveground of what plants have roots reaching the water in the cenotes there is no way to know what caves must be explored to find more roots. Moreover, the access to many cenotes is so difficult that only highly qualified divers can reach them. Consequently, a systematic exploration of the underground aquifer to locate several roots of the same species is very complicated.

## Supplementary Information

**Additional file 1: Figure S1.** Beta diversity analysis.

**Additional file 2: Table S1.** Low abundance phyla list.

**Additional file 3: Table S2.** PCR conditions for DNA amplification.** Table S3.** Plant species identification, GenBank accesion numbers.

## Data Availability

Plant species DNA barcodes are available at the website of GenBank for the *rbcL*, *matK* and *psbA*-*trnH* regions under the numbers: *Ficus obtusifolia*, MK643034, MK643037 and MK643040; *Gliricidia sepium*, MK643035, MK643038 and MK643041; *Trichilia hirta*, MK643036, MK643039 and MK643042. 16*S r*RNA sequences are available at the NCBI website under the BioProject database number PRJNA559701 (https://www.ncbi.nlm.nih.gov/bioproject/?term=PRJNA559701).
