# Supplementary figures and images for: Distinctive prokaryotic microbiomes in sympatric plant roots from a Yucatan cenote
Source: BMC Res Notes. 2021 Sep 7;14:333. doi: 10.1186/s13104-021-05746-x (PMC8424917; doi:10.1186/s13104-021-05746-x)

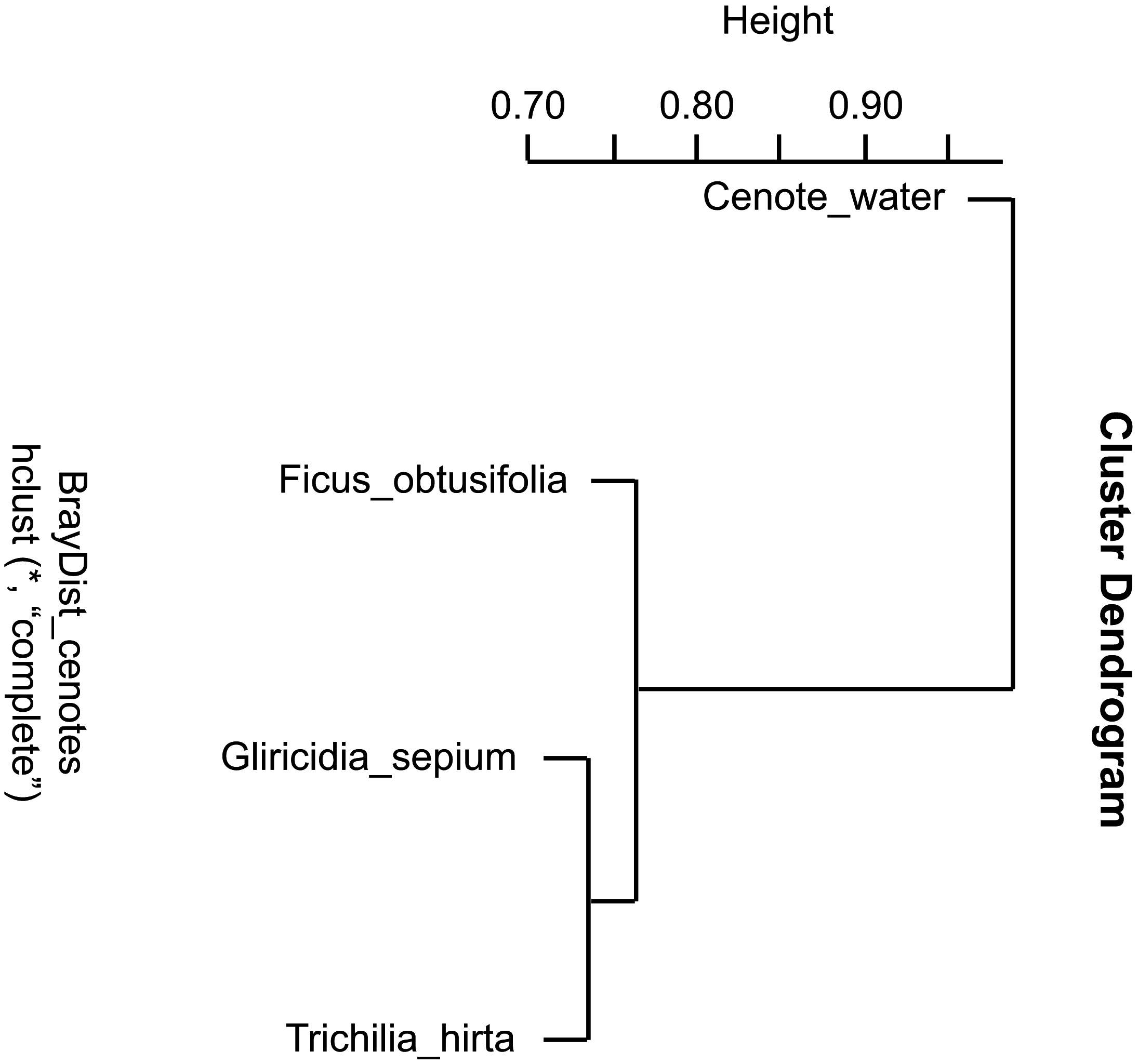

Supplement: Supplementary file 1 — Additional file 1: Figure S1. Beta diversity analysis. [file 13104_2021_5746_MOESM1_ESM.jpg]
