# Supplementary material for: Distinctive prokaryotic microbiomes in sympatric plant roots from a Yucatan cenote
Source: BMC Res Notes. 2021 Sep 7;14:333. doi: 10.1186/s13104-021-05746-x (PMC8424917; doi:10.1186/s13104-021-05746-x)
Supplement: Supplementary file 3 — Additional file 3: Table S2. PCR conditions for DNA amplification. Table S3. Plant species identification, GenBank accesion numbers. [file 13104_2021_5746_MOESM3_ESM.pdf]

Amplified region	Primer sequence (5‘–3‘)	PCR conditions
RuBisCo Large subunit<br>Levin 2003<br>Kress 2007	*rbcLa_F:*<br>ATGTCACCACAAACAGAGACTAAAGC<br>*rbcLa_R:*<br>GTAAAATCAAGTCCACCRCG	94°C 2 min;<br>29 cycles: 94°C 1 min,<br>48°C 1 min, 72°C 2.5 min;<br>72°C 7 min
Non-ribosomal plastid-encoded RNA binding protein<br>CCDB protocols	*matK_1RKIM_F:*<br>ACCCAGTCCATCTGGAAATCTTGGTTC<br>*matK_3FKIM _R:*<br>CGTACAGTACTTTTGTGTTTACGAG	94°C 2 min;<br>30 cycles: 94°C 30 s, 48°C 40 s, 72°C 40 s;<br>72°C 5 min
Non-ribosomal plastid-encoded RNA binding protein<br>Cuenoud 2002	*matK_390F:*<br>CGATCTATTCATTCAATATTTC<br>*matK_1326R:*<br>TCTAGCACACGAAAGTCGAAGT	94°C 2 min;<br>35 cycles: 94°C 30 s, 50°C 40 s, 72°C 40 s;<br>72°C 5 min
Plastid intergenic spacer<br>Sang 1997<br>Tate 2003	*psbA3_F:*<br>GTTATGCATGAACGTAATGCTC<br>*trnHf_05_R:*<br>CGCGCATGGTGGATTCACAATCC	94°C 2 min;<br>29 cycles: 94°C 30 s, 52°C 30 s, 72°C 1 min;<br>72°C 7 min

Table S2. PCR conditions for cenote roots and water DNA amplification.

Plant species	*rbcL*	*matK*	*psbA-trnH*
Gliricidia sepium (*Fabaceae*)	MK643035	MK643038	MK643041
Trichilia hirta (*Meliaceae*)	MK643036	MK643039	MK643042
Ficus obtusifolia (*Moraceae*)	MK643034	MK643037	MK643040

Table S3. GenBank accession numbers for root DNA.
